# *Schistosoma mansoni* Eggs in Spleen and Lungs, Mimicking Other Diseases

**DOI:** 10.1371/journal.pntd.0003860

**Published:** 2015-07-23

**Authors:** Federico Gobbi, Giulia Martelli, Luciano Attard, Dora Buonfrate, Andrea Angheben, Valentina Marchese, Laura Bortesi, Maria Gobbo, Elisa Vanino, Pierluigi Viale, Zeno Bisoffi

**Affiliations:** 1 Center for Tropical Diseases, Ospedale Sacro-Cuore Don Calabria, Negrar, Verona, Italy; 2 Infectious Diseases Unit, Department of Medical and Surgical Sciences, Ospedale S. Orsola-Malpighi, Alma Mater Studiorum University of Bologna, Bologna, Italy; 3 Acute and Chronic Viral Hepatitis Department, Seconda Università degli Studi di Napoli, Naples, Italy; 4 Department of Pathology, Ospedale Sacro-Cuore Don Calabria, Negrar, Verona, Italy; National Institute of Parasitic Diseases, Chinese Center for Diseases Control and Prevention, China

## Case Presentation

### Case 1

**Fig 1:**
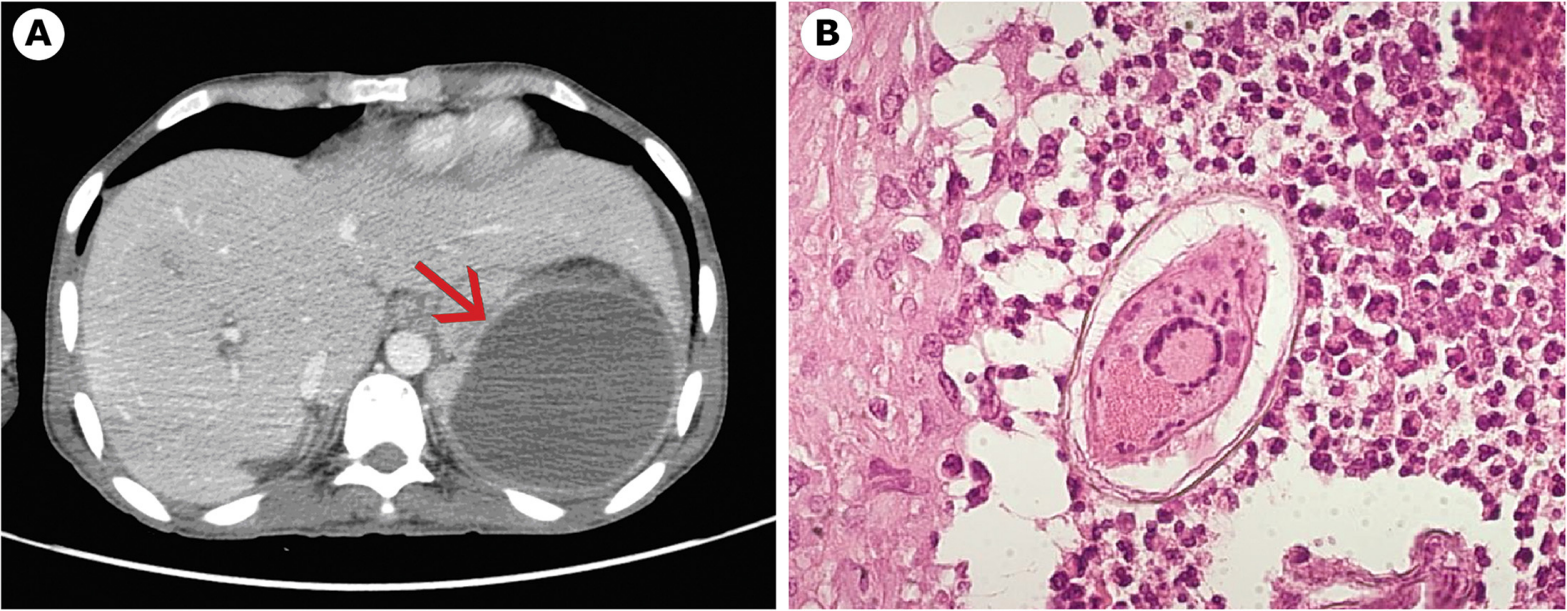
Case 1. a) Splenic lesion at CT scan. b) *Schistosoma mansoni* egg in spleen tissue.

A 25-year-old migrant presented to the Unit of Infectious Diseases of Bologna in October 2013. After leaving Senegal in 2009, he stayed in Libya for two years before reaching Italy by boat in 2011. In August 2013, he was involved in a road accident, reporting a fracture of the pelvis and a traumatic bowel perforation. A rectal resection with colostomy was carried out, and external osteosynthesis was done. After the surgical intervention, sepsis appeared: fever >38°C, tachypnea, WBC 28,000/μl, and procalcitonin 2.3 nanogr/ml (normal <0.5). An abdominal CT scan showed portal vein thrombosis and multiple embolic infarctions involving the lungs, kidneys, and spleen. A carbapenemase-resistant *Klebsiella pneumoniae* (KPC) strain was identified in the blood culture, in the rectal swabs, in the colostomy, and in the sputum specimens; an intravenous antibiotic treatment with meropenem, colistin, tigecycline, and rifampin was promptly started. The patient’s conditions slightly improved, but a further abdominal CT scan revealed a fluid intrasplenic lesion (Fig 1A).

Suspecting a massive splenic hematoma, a splenectomy was carried out on November 2013. An abundant purulent exudate was found in the spleen; the histologic examination revealed several *Schistosoma mansoni* eggs (Fig 1B). Serology for *S*. *mansoni* (enzyme immunoassay [EIA]) resulted positive (37 U/mL, negative <12), and *S*. *mansoni* eggs were detected in stool specimens too. The blood tests showed hypereosinophilia (2,510 eosinophils/μl) and raised IgE (7,110 IU/mL). The patient was treated with praziquantel (40 mg/kg/day, divided in two doses, for 3 d). After two weeks, eosinophils decreased, and no more *S*. *mansoni* eggs were found in the stool; his condition promptly improved and antibiotic therapy was stopped. Six months after the accident, he successfully underwent surgical restoration of intestinal continuity. Rectal swab test for KPC resulted negative.

### Case 2

**Fig 2:**
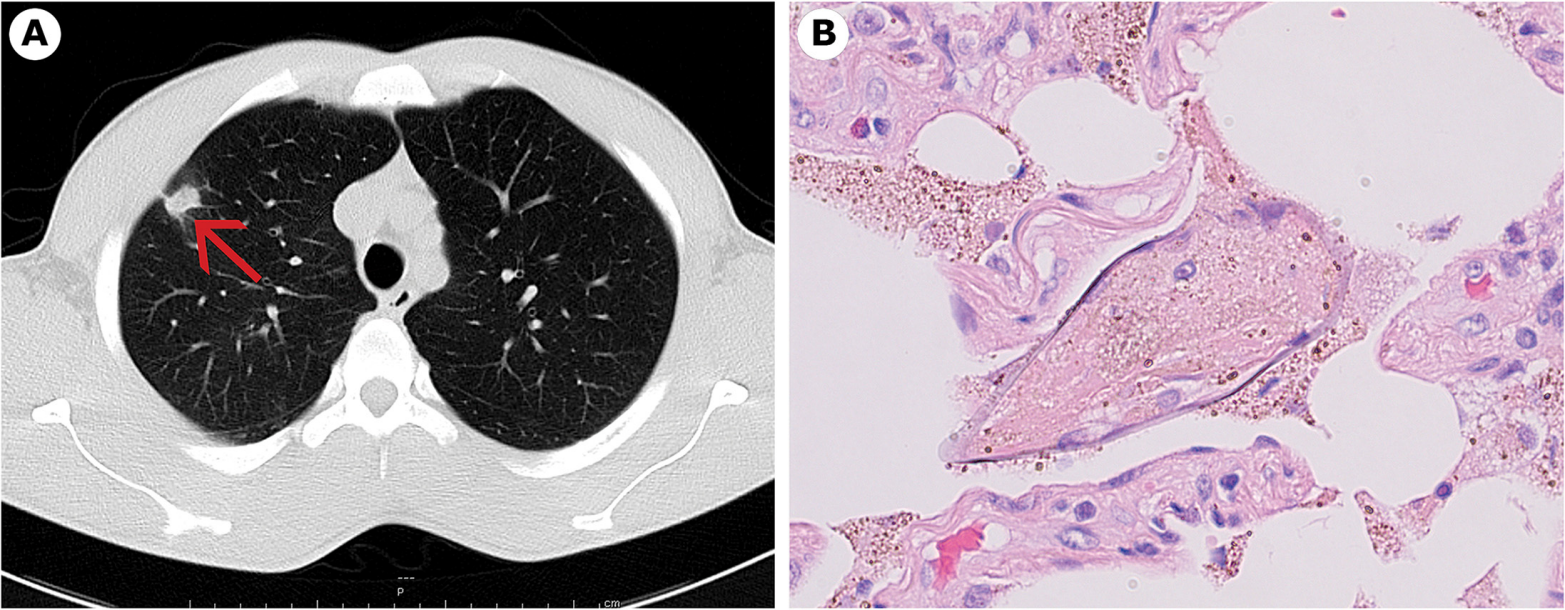
Case 2. a) Pulmonary nodule at CT scan. b) *S. mansoni* egg in lung tissue.

A 27-year-old migrant presented to the Center for Tropical Diseases (CTD), Negrar, Verona, in May 2014, complaining of abdominal pain for 2 mo. After leaving Mali in 2009, he stayed in Libya for five years before reaching Italy by boat in February 2014. The blood tests showed 690 eosinophils/μl, further increased up to 1,020/μl; C reactive protein and erythrocyte sedimentation rate tested normal. The IgE was elevated (4,950 IU/mL). The quantiferon tuberculosis (TB) test was negative and so were anti-HIV and anti-HCV antibodies. A chronic hepatitis B infection was diagnosed. Serology for *S*. *mansoni* (ELISA) resulted positive (Optical Density 4.07, negative <0.9), and *S*. *mansoni* eggs were detected in stool specimens. The patient was treated with praziquantel (40 mg/kg/day, divided in two doses, for 3 d). Abdominal echography revealed signs of cirrhosis and moderate splenomegaly. A liver biopsy demonstrated periportal “Symmer’s pipestem” fibrosis and the presence of *S*. *mansoni* eggs. Chest X-ray showed a small pulmonary nodule in the right lung. A pulmonary CT scan detected multiple nodules between 7 and 13 mm of diameter (Fig 2A).

A biopsy allowed us to remove the bigger nodule (13 mm diameter), located in the right lung, close to the pleura. Histological examination of the nodule revealed several *S*. *mansoni* eggs (Fig 2B) surrounded by histiocytes. Meanwhile, reverse transcription PCR (RT-PCR) resulted positive for *Mycobacterium tuberculosis* on two sputum samples, and a specific treatment for tuberculosis was started.

### Case Discussion

Usually, eggs of *S*. *mansoni* are found in stool, the intestinal tract, and the liver, rarely in the central nervous system (CNS) [1]. Ectopic sites of *S*. *mansoni* are reported in the literature, but almost all of them were found by autopsy. Ectopic sites are defined as adult worms and/or eggs outside the portal-mesenteric system, that is, excluding the liver and intestine [2]. Gonçalves et al., in their series of 1,863 consecutive autopsies carried out in Belo Horizonte, Brazil, reported 313 cases (16.8%) of *S*. *mansoni* infection, with eggs found in almost all organs; ninety-three cases with schistosomiasis (29.7%) had eggs in the lungs, while one case (0,3%) had eggs in the spleen. Other organs involved were the pancreas (6.4%), brain (4.8%), lymph nodes (3.8%), testicles (3.2%), kidney (2.9%), oesophagus (1.0%), gall bladder (1.0%), heart (1.0%), stomach (0.3%), spinal cord (0.3%), ovary (0.3%), pleura (0.3%), prostate (0.3%), and eye (0.3%) [2]. According to the authors, ectopic locations probably derive from a high parasite burden, causing portal hypertension and subsequent embolization of the parasite eggs through the collateral portal-systemic circulation [2]. This theory could explain a diffuse homogeneous presence of *S*. *mansoni* eggs in the different organs, but not the finding of a mass as reported in our two cases. Faust in 1947 reported that adult worms, too, were occasionally discovered [3]: a male of *S*. *haematobium* in the circumflex branch of the coronary artery [4], and a pair of male and female worms with degenerating eggs in a branch of the superior ophthalmic vein, causing a palpebral tumor [5]. Brumpt, in 1949, reported that females of *S*. *mansoni* can be found, and lay eggs, in different organs including the lungs [6].

Faust analysed five possible explanations of ectopic lesions in schistosomiasis [3]: the first theory, that the metacercariae develop to adult worms near the sites of penetration into the skin, was considered not consistent with critical studies on the development of schistosomes in the body of the definitive host; the second theory, a patent foramen ovale, implied a highly improbable combination of circumstances; the third theory, that the eggs may escape through the pulmonary capillaries and be deposited in distant arterioles, was discarded because of the characteristic disposition of eggs in nests or aggregates, and the tissue reaction around the venules rather than arterioles. He considered more probable the fourth theory, that adult worms may travel against venous blood flow into collateral vessels and, on reaching the end venules, deposit their eggs, and the fifth theory, that the vertebral venous system provides a natural, valveless, intercommunicating channel from portal and caval veins to all parts of the body [3].

A common feature of our two cases was the presence of comorbidities: in case 1, a sepsis due to KPC and in case 2, a pulmonary TB. Interestingly, a case of pulmonary schistosomiasis characterized by a lower lobe lung mass associated with pulmonary TB was recently reported [7]: the species involved was a *S*. *japonicum*, and the eggs were found at postmortem examination [7].

In the literature, an association between *S*. *mansoni* and bacteremia is reported: the bacteria more frequently associated are *Salmonella*, *Escherichia coli*, and *Salmonella aureus* [8,9]. Several pathogenic mechanisms have been proposed to explain this association: from an enhanced bacterial adhesion to granulomata to an inhibition of Th1 immune response [8,10]. Schistosomiasis is also reported to be a predisposing factor for pyogenic liver abscess [8,10,11]. In one of the case reports, the liver biopsy showed a great number of *S*. *mansoni* eggs in the hepatic tissue [11].

Unnecessary surgical procedures for unrecognized parasitic diseases have been reported both for the lungs and the spleen. According to a case report from Martinique reported in 1988, an open chest lung biopsy was performed in a female patient on suspicion of Carrington’s disease, resulting in a case of pulmonary *S*. *mansoni* bilharziasis [12]. In the United States (2008) and in Japan (2007), two patients with pulmonary nodules were submitted, respectively, to a fine-needle aspiration biopsy and to a right middle and lower lobectomy; the histological exam revealed in both cases the presence of *Paragonimus* [13,14]. Concerning unnecessary splenectomy, performed under suspicion of malignant lymphoproliferative disorders, several examples have been reported for different parasitic diseases. Three patients developed malaria after surgery, with a final diagnosis of hyperreactive-malarial splenomegaly (HMS) [15,16]. In two cases, the true cause of splenomegaly was an unrecognized visceral leishmaniasis [17,18]. In two more cases, histology of the spleen revealed multiple granulomata around *Loa loa* microfilariae [19]. The description of the latter, published cases, was crucial to spare an unnecessary splenectomy to a further patient seen at CTD, Negrar by some of us [20]. Besides a clinical suspicion of loiasis (hypereosinophilia, Calabar swelling), not confirmed by laboratory identification, the latter patient also presented multiple hypoechogenic lesions of the spleen that completely disappeared (along with the eosinophilia and the other clinical signs) a few weeks after presumptive treatment of *L*. *loa* infection with diethylcarbamazine DEC [20]. As for schistosome, to our knowledge, our case 1 is the first description of *S*. *mansoni* eggs in the spleen tissue of a living patient.

We treated both patients with a three day course of praziquantel (40 mg/kg/day, divided in two doses), which differs from WHO recommendations (40mg–60mg/kg body weight as a single dose) [21]. In a meta-analysis carried out by Zwang and Olliaro [22], the cure rate of a single dose of praziquantel was 76.7% and, considering that our patients were not at risk for reinfection, we wanted to increase the odds of cure.

### Conclusions

In the global village, clinicians should open their “clinical landscape” to a number of diseases they have little, if any, familiarity with. Moreover, some of them may present with uncommon, atypical presentations that may be mistaken for other diseases. With this possibility in mind, an accurate clinical and epidemiological history, along with appropriate laboratory exams, can help the clinician to make the appropriate decision while avoiding unnecessary invasive and potentially dangerous procedures. In particular, the clinicians should consider schistosomiasis due to *S*. *mansoni* in case of a patient coming from sub-Saharan Africa, the east coast of South America between Venezuela and Brazil, the Caribbean, Egypt, and the Arabian peninsula.

### Ethics Statement

The two patients gave written informed consent for the publication of these case reports.

## Key Learning Points

- Ectopic eggs of *S*. *mansoni* can be found in different organs including the lungs and spleen.
- Schistosomiasis should be considered in “the clinical landscape” in case of travellers coming from endemic countries with hypereosinophilia, even if the presentation is not typical.
- Unnecessary surgical procedures for unrecognized parasitic diseases can be avoided by an accurate clinical and epidemiological history, along with appropriate laboratory exams.
- In the literature, an association between *S*. *mansoni* and bacteremia is reported: the bacteria more frequently associated are *Salmonella*, *E*. *coli* and *S*. *aureus*.
